# *Bassia indica* Attenuates Cardiotoxicity in a Rat Model via Anti-Inflammatory, Antioxidant, and Keap1/Nrf2 Modulation

**DOI:** 10.3390/ph18121907

**Published:** 2025-12-18

**Authors:** Fayyaz Anjum, Saad Touqeer, QurratUlAin Jamil, Ayesha Rida, Hafiz Muhammad Zubair, Adeel Sarfraz, Saleh Alfuraih, Waad Alrohily, Ali F. Almutairy, Ashfaq Ahmad, Mohammed Aufy, Shahid Muhammad Iqbal

**Affiliations:** 1 Department of Pharmacology, Faculty of Pharmacy, The Islamia University of Bahawalpur, Bahawalpur 63100, Pakistan; fayyaz.anjum@iub.edu.pk (F.A.);; 2 College of Pharmacy, Al Ain University, Abu Dhabi 112612, United Arab Emirates; 3 AAU Health and Biomedical Research Center, Al Ain University, Abu Dhabi 112612, United Arab Emirates; 4 Department of Pharmacy Practice, Faculty of Pharmacy, The Islamia University of Bahawalpur, Bahawalpur 63100, Pakistan; 5 Post Graduate Medical College, Faculty of Medicine & Allied Health Sciences, The Islamia University of Bahawalpur, Bahawalpur 63100, Pakistan; 6 Department of Anatomy and Histology, Faculty of Veterinary and Animal Sciences, The Islamia University of Bahawalpur, Bahawalpur 63100, Pakistan; 7 Department of Pharmacology and Toxicology, College of Pharmacy, Northern Border University, Rafha 76313, Saudi Arabia; saleh.alforih@nbu.edu.sa; 8 Department of Pharmacy Practice, College of Pharmacy, Taibah University, Medina 52571, Saudi Arabia; 9 Department of Pharmacology and Toxicology, College of Pharmacy, Qassim University, Buraydah 51452, Saudi Arabia; 10 Department of Pharmacy Practice, College of Pharmacy, University of Hafr Al Batin, Hafr Al Batin 39524, Saudi Arabia; 11 Division of Pharmacology and Toxicology, University of Vienna, UZA II, Josef-Holaubek-Platz 2, A-1090 Vienna, Austria

**Keywords:** cardioprotective, inflammation, Nrf2 signaling, antioxidants, *Bassia indica*

## Abstract

**Background**: Drug-induced cardiotoxicity is a primary concern in clinical practice, especially in the context of oxidative stress induced by anti-cancer, antiviral, and antidiabetic drugs. Several strategies are devised to limit cardiotoxicity, which are supportive and provide symptomatic relief. This highlights the need to develop cardioprotective agents that circumvent the oxidative stress. *Bassia indica* is a cardiotonic plant with antioxidant properties traditionally used in Africa, South Asia, and China. We investigated its cardioprotective effects against doxorubicin-induced cardiotoxicity (DIC). **Methods**: *B. indica* extract (BiE) was analyzed by GC-MS and HPLC. Several antioxidant assays, including DPPH, FRAP, CUPRAC, NO, and H_2_O_2_ scavenging, were performed. In vivo attenuation of DIC was assessed in a rat model. **Results**: BiE contained several bioactive flavonoids, including 2-methoxy-4-vinylphenol, ferulic acid, gallic acid, kaempferol, and coumaric acid. Antioxidant assays demonstrated potent free-radical scavenging and antioxidant activity of BiE, providing mechanistic evidence for its in vivo amelioration of DIC. BiE treatment reduced myocardial oxidative stress by increasing endogenous antioxidant levels (*p* < 0.01), including SOD, CAT, and GSH. It upregulated Nrf2 and lowered Keap1 levels. This was also reflected in the restoration of cardiac tissue architecture and modulation of inflammatory markers, including IL-1β and TNF-α (*p* < 0.01). Cardiac tissue biomarkers were also improved. **Conclusions**: These findings conclude that BiE exerts cardiac protection by reducing oxidative stress and inflammation through modulation of the Keap1/Nrf2 pathway and decreasing the expression of IL-1β and TNF-α.

## 1. Introduction

Drug-induced cardiotoxicity (DICT) is a significant challenge in clinical practice, which can present both structural and functional abnormalities. These include arrhythmias, conduction abnormalities, myocarditis, thrombosis, heart failure, and myocardial infarction (MI) [1]. Several clinically used drugs, for instance, anti-cancer (doxorubicin and cisplatin), antidiabetic (pioglitazone and rosiglitazone), and antivirals (zidovudine), have serious cardiac toxicity. Heart tissues have a limited antioxidant capacity, so drugs that induce oxidative stress and inflammation can cause cardiac cell damage, apoptosis, and necrosis [2,3]. There are several mechanisms of DICT however it is believed that during metabolism, reactive metabolites interact with molecular oxygen and generate ROS (reactive oxygen species) such as hydroxyl radicals (HO•), O_2_•- (superoxide anions), H_2_O_2_ (hydrogen peroxide), and NO• (nitric oxide)—which alongside impairing the mitochondrial function, also exhaust antioxidant defenses, and increase the lipid peroxidation. The tissue levels of endogenous antioxidant enzymes, such as GSH, glutathione peroxidase (GPx), CAT, and SOD, are notably reduced during this process. Elevated ROS disrupts normal cellular signaling, including B-cell lymphoma-2 (Bcl-2), Keap1/Nrf2, nuclear factor kappa-light-chain-enhancer of activated B cells (NF-κB), protein kinase C (PKC), mitogen-activated protein kinase (MAPK), phosphatidylinositol 3-kinase/protein kinase B (PI3K/Akt), p38, and c-Jun N-terminal kinase (JNK) pathways. Particularly, the downregulation of Bcl-2 (anti-apoptotic) protein and upregulation of caspase-3 trigger cardiomyocyte apoptosis. This oxidative imbalance promotes inflammation by creating a pro-inflammatory microenvironment. Activation of NF-κB leads to the release of cytokines such as TNF-α, IL-1β, IL-6, and IL-8, and to the enhancement of cyclooxygenase-2 (COX-2), 5-lipoxygenase (5-LOX), inducible nitric oxide synthase (iNOS), chemokines, and adhesion molecules. These changes intensify cardiac injury and contribute to the development of heart failure, coronary artery disease, and impaired ventricular function [4].

Reducing oxidative stress and suppressing inflammatory cytokines after cardiac injury has been shown to mitigate DICT [5]. The primary pathway regulating oxidative stress in cardiac tissue is the Keap-1/Nrf2 pathway. Keap-1 governs the production of the antioxidant transcription factor Nrf2, an oxidation-reduction-sensitive transcription factor that promotes the expression of genes involved in cell survival, controls mitochondrial function, stimulates the expression of endogenous antioxidant enzymes, and consequently decreases oxidative damage. Under high oxidative stress, Nrf2 escapes the keap-1-mediated inhibition and increases the transcription of antioxidant enzymes [2,6]. Management of DICT is mainly supportive and symptomatic. When a patient shows abnormal left ventricular ejection fraction or elevated cardiac biomarkers, initiation of angiotensin-converting enzyme inhibitors (ACEIs) or angiotensin receptor blockers (ARBs) is recommended. However, these therapies come with potential drawbacks, particularly in cancer patients who may already exhibit abnormal heart rate or blood pressure due to disease manifestations or prescribed medicines. Dexrazoxane has emerged as a cardioprotective agent that mitigates the anthracycline-induced cardiac injury, but its limited availability and safety concerns constrain its use [7]. Limited therapeutic options and identification of oxidative stress as a key pathway in DICT encourage the investigation of antioxidants as cardioprotective agents. Many established antioxidant agents, such as vitamins C and E, and natural compounds that can upregulate the Keap1/Nrf2 pathway, have shown evidence of mitigating the DICT [8]. In this regard, medicinal plants offer a unique advantage due to their diverse antioxidant and anti-inflammatory activities. They directly scavenge the ROS and RNS and upregulate cellular defense mechanisms against oxidative stress. For instance, *Salviae miltiorrhizae* protects against DIC by modulating inflammatory and oxidative stress signaling pathways [9]. Geraniol, isolated from lemon grass, has been shown to upregulate the Nrf2 and increase levels of SOD, GST, and GSH in DIC [10]. Similarly, *Vaccinium corymbosum* increased GSH and SOD levels and upregulated Nrf2 and Sirtuin 2 [11].

*Bassia indica* (Wight) A.J. Scott (synonyms: *Kochia indica*) belongs to the Amaranthaceae family and is commonly found in Africa, Asia, and Mediterranean countries [12,13]. It has been traditionally used to treat cardiac and inflammatory conditions. In Pakistan [14,15], Tunisia [16], Egypt [17], and Saudi Arabia [18], preparations from the whole plant, decoctions, and its oil are used as cardiotonics. Previous studies have reported that *B. indica* contains several bioactive phytochemicals, including kaempferol, ferulic acid, gitoxigenin, azafrin, and betulin, which modulate oxidative stress and inflammatory signaling pathways and exhibit cardioprotective activities [19]. Kaempferol, azafrin, and ferulic acid upregulate the Nrf2/ARE pathway and mitigate inflammation by inhibiting NF-κB and COX-2 [20,21,22]. Betulin also protects against myocardial damage by inhibiting the NF-κB, Bcl-2-associated X (Bax), and caspase-3 signaling pathways [23]. Our previous work demonstrated that *B. indica* is safe up to 2 g/kg when administered orally [19] and protects against stress-induced myocardial infarction by inhibiting COX-2 and downregulating inflammatory mediators, including IL-1β, TNF-α, NF-κB, and Bax [24]. We observed strong in vitro antioxidant activity in *B. indica*. Therefore, in this study, we investigated whether antioxidant activity provides cardioprotection against DIC and explored the possible molecular mechanisms underlying its protective action.

## 2. Results

### 2.1. Extract Preparation and Fractionation

The extraction process produced 264 g of BiE, corresponding to an 8.8% yield. Further fractionation of BiE showed notable differences in extract distribution, including n-hexane (1%), dichloromethane (1.5%), ethyl acetate (2%), n-butanol (3%), and aqueous (10.5%).

### 2.2. GC-MS of BiE

GC-MS analysis showed that BiE has several phytochemicals with the highest abundance observed for n-hexadeconic acid, followed by 10-*(E)*, 12-*(Z)*-octadecadienoic acid, *(E)*-4-(3-hydroxyprop-1-en-1-yl)-2-methoxyphenol, and 2-methoxy-4-vinylphenol. The GC-MS chromatogram of BiE is shown in Supplementary Figure S1, and the identified compounds, determined by comparing spectral patterns with the NIST20.L library and having ≥90 qual factors, are given in Table 1.

### 2.3. HPLC Assay

Several bioactive phenolic acids and flavonoids were identified in HPLC analysis of BiE. These compounds are given in Table 2, while HPLC chromatograms are provided in Figure S2a,b.

### 2.4. Antioxidant Activity of BiE

DPPH analysis of BiE showed that the ethyl acetate fraction was the most potent, with an IC_50_ of 12.6 ± 2.6 µg/mL, closely matching the activity of ascorbic acid (11.9 ± 3.8 µg/mL). In comparison, the n-hexane fraction was least potent with an IC_50_ of 203.6 ± 50.3 µg/mL. In the FRAP assay, BiE exhibited the highest activity with an IC_50_ of 119.6 ± 5.2 µM AAE/g BiE, followed by ethyl acetate 110.25 ± 14.63 µM AAE/g BiE. NO radical scavenging assay showed that n-butanol was most potent with an IC_50_ value of 0.17 ± 0.008 mg/mL, while ascorbic acid had an IC_50_ value of 0.46 ± 0.04 mg/mL. The CUPRAC assay determined the reducing potential of BiE, and the ethyl acetate fraction showed the highest reducing activity (126.96 ± 0.23 mg AAE/g BiE). The H_2_O_2_ free radical scavenging assay showed that the dichloromethane fraction had the highest activity, with an IC_50_ value of 133.1 ± 6.6 µg/mL, which was close to the ascorbic acid value of 128.7 ± 7.8 µg/mL. IC_50_ values of all fractions are shown in Table 3.

### 2.5. BiE Treatment Improves Cardiac Markers

**Figure 1:**
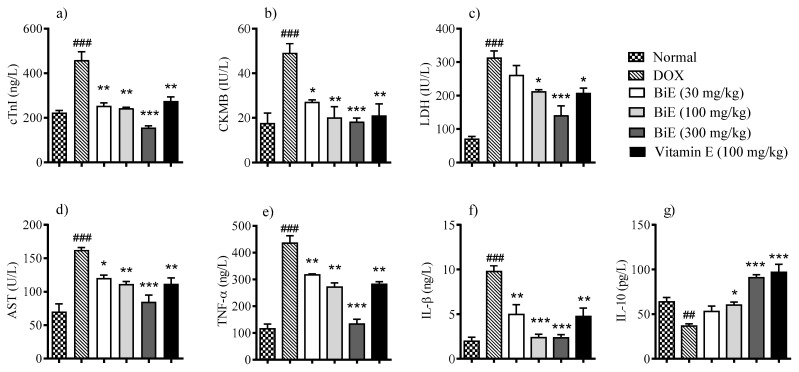
BiE and vitamin E effects on relative serum concentrations of (**a**) cTnI, (**b**) CK-MB, (**c**) LDH, (**d**) AST, (**e**) TNF-α, (**f**) IL-1β, and (**g**) IL-10 in doxorubicin-induced cardiotoxicity. Data (mean ± SEM, n = 5) were analyzed using one-way ANOVA with Tukey’s post hoc test. Comparison between the DOX and normal groups is indicated by hash symbols, with significance levels shown as ^##^ = *p* < 0.01, ^###^ = *p* < 0.001. In contrast, differences between the BiE or vitamin E groups and the DOX group are indicated by asterisks with significance levels denoted as * = *p* < 0.05, ** = *p* < 0.01, and *** = *p* < 0.001.

Doxorubicin significantly (*p* < 0.01) increased cTnI serum level 459 ± 37 ng/L in the DOX group compared to 224 ± 8.7 ng/L in the normal group, indicating myocardial injury. Groups treated with different BiE dosses (30, 100 and 300 mg/kg) and vitamin E (100 mg/kg) resisted the cellular injury and decreased serum levels of cTnI to 254 ± 13 ng/L (*p* < 0.01), 243 ± 5.2 ng/L (*p* < 0.01) 156 ± 8.0 ng/L (*p* < 0.001) and 275 ± 19 ng/L (*p* < 0.01), respectively (Figure 1a). A similar pattern was observed in serum CK-MB and LDH levels. CK-MB (49 ± 4.1 IU/L) and LDH (314 ± 20 IU/L) levels were increased (*p* < 0.001) in the DOX group as compared to the normal group (18 ± 4.4 and 72 ± 6.3 IU/L, respectively). A dose-dependent decrease of 27 ± 0.91 IU/L, 20 ± 4.9 IU/L, and 18 ± 1.6 IU/L in CK-MB serum values was observed with the treatment of 30, 100, and 300 mg/kg BiE (Figure 1b). BiE treatment also decreased the LDH levels. Maximum reduction to 141 ± 28 IU/L, *p* > 0.01, was observed at 300 mg/kg (Figure 1c). Vitamin E administration also significantly lowered the CK-MB (*p* < 0.01) and LDH (*p* < 0.05) levels. Serum AST levels were increased to 162 ± 3.7 U/L in the DOX group compared to the normal group, 70 ± 11 U/L, *p* < 0.001. BiE and vitamin E treatment prevented the increase in serum AST levels (Figure 1d).

### 2.6. BiE Treatment Improves Inflammatory Mediators

Pro-inflammatory markers TNF-α and IL-1β were elevated (*p* < 0.001) in the DOX group, at 435 ± 28 ng/L and 9.8 ± 0.63 ng/L, respectively, compared with the normal control group, at 115 ± 18 ng/L and 2.0 ± 0.45 ng/L, respectively. BiE treatment prevented the increase in TNF-α and IL-1β levels in a dose-dependent manner, with the maximum reductions observed with BiE 300 mg/kg: 133 ± 18 ng/L and 2.4 ± 0.37 ng/L, respectively. Similarly, vitamin E treatment also significantly reduced these pro-inflammatory markers (*p* < 0.01), as shown in Figure 1e,f. Anti-inflammatory marker IL-10 was decreased to 37 ± 2.4 pg/L in the DOX group. At the same time, BiE treatment increased serum IL-10, with a maximum increase of 91 ± 3.3 pg/L observed in the 300 mg/kg-treated group, which was significant (*p* < 0.001) compared to the DOX group. Vitamin E treatment also increased (*p* < 0.001) the IL-10 as compared to the diseased group (Figure 1g).

### 2.7. BiE Treatment Improves Oxidative Stress Markers

**Figure 2:**
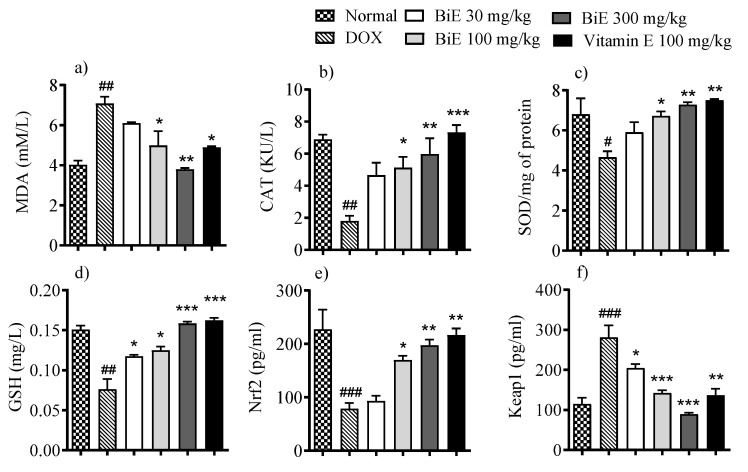
BiE and vitamin E treatment effects on tissue concentrations of (**a**) MDA, (**b**) CAT, (**c**) SOD, (**d**) GSH, (**e**) Nrf2, and (**f**) Keap1 in doxorubicin-induced cardiotoxicity. Data (mean ± SEM, n = 5) were analyzed using one-way ANOVA with Tukey’s post hoc test. Comparisons between the DOX and Normal groups are indicated by hash symbols, with significance levels shown as ^#^ = *p* < 0.01, ^##^ = *p* < 0.01, and ^###^ = *p* < 0.001. In contrast, differences between the BiE or vitamin E groups and the DOX group are indicated by asterisks, with significance levels denoted as * = *p* < 0.05, ** = *p* < 0.01, and *** = *p* < 0.001.

Oxidative markers in heart tissue showed alterations after doxorubicin administration. MDA tissue levels were elevated (7.1 ± 0.34 µM/L) in the DOX group compared to the normal group (4.0 ± 0.21 µM/L), indicating damage to myocyte cell membranes. BiE treatment decreased MDA concentrations with increasing dose, with a maximum effect observed at 300 mg/kg (3.8 ± 0.06 µM/L), comparable to that of vitamin E (Figure 2a). Doxorubicin also reduced the other oxidative markers, including SOD (*p* < 0.05), catalase (*p* < 0.01), and GSH (*p* < 0.01) in the DOX group compared to the normal control group. Groups treated with BiE and vitamin E restored these markers, indicating alleviation of doxorubicin-induced oxidative stress (Figure 2b–d). Keap1 levels increased to 282 pg/L in the DOX group compared with the normal group (115 ± 16 pg/L). BiE treatments reduced Keap1 levels, with the most significant reduction observed in the 300 mg/kg group (90 ± 3.8 pg/L; *p* < 0.001). Vitamin E treatment also reduced Keap1 levels (137 ± 17 pg/L) significantly (*p* < 0.01). This also affected downstream Nrf2 levels, which were reduced (*p* < 0.01) in the DOX group compared with the normal group. A dose-dependent increase in Nrf2 levels was seen in BiE pre-treated groups and the vitamin E-treated group, as shown in Figure 2e,f.

### 2.8. mRNA Expressions of Inflammatory Interleukins and the Keap1/Nrf2 Pathway

**Figure 3:**
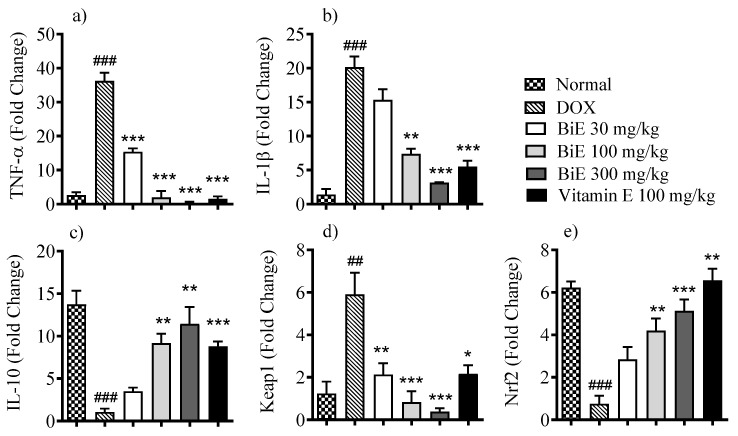
Relative mRNA levels of (**a**) TNF-α, (**b**) IL-1β, (**c**) IL-10, (**d**) Keap1, and (**e**) Nrf2 in cardiac tissue after BiE and vitamin E treatment in DOX-induced cardiotoxicity. Gene expression was normalized to GAPDH (2^−ΔΔCt^). Data (mean ± SEM, n = 3) were analyzed using one-way ANOVA with Tukey’s post hoc test. Comparisons between the DOX and Normal groups are indicated by hash symbols, with significance levels shown as ^##^ = *p* < 0.01, ^###^ = *p* < 0.001. In contrast, differences between the BiE or vitamin E groups and the DOX group are indicated by asterisks, with significance levels denoted as * = *p* < 0.05, ** = *p* < 0.01, and *** = *p* < 0.001.

The effects of BiE treatment on mRNA expression levels of various inflammatory and oxidative stress markers were assessed to determine the roles of inflammation and oxidative stress regulation. Melt-curve analyses confirmed single-peak amplification for each target. Amplification efficiencies ranged between 93 and 105%, within acceptable limits for comparative Ct analysis. The mRNA levels of pro-inflammatory markers, i.e., TNF-α and IL-1β, were increased in the DOX group, with 36 ± 2.4- and 20 ± 1.6-fold increases, respectively. In contrast, anti-inflammatory interleukin IL-10 levels decreased by 1.1 ± 0.40-fold compared with the normal group (Figure 3). BiE treatment prevented the inflammation caused by DOX in a dose-dependent manner, with the highest effects seen at 300 mg/kg, as mRNA expression of TNF-α (0.38 ± 0.32-fold change; *p* < 0.001), IL-1β (3.2 ± 0.07-fold change; *p* < 0.001), and IL-10 (11 ± 2.0-fold change; *p* < 0.001) was observed. Vitamin E also decreased TNF-α (*p* < 0.001), IL-1β (*p* < 0.01), and increased IL-10 (*p* < 0.01) significantly (Figure 3a–c). BiE treatments also modulated the Keap1/Nrf2 signaling pathway. It decreased the mRNA expression of Keap1 at a 300 mg/kg dose (0.38 ± 0.16-fold change; *p* < 0.001) compared to the DOX group (5.9 ± 1.0-fold change, *p* < 0.001) and increased Nrf2 mRNA expression (5.1 ± 0.54-fold change; *p* < 0.01), compared to the DOX group (0.76 ± 0.38-fold change). The BiE 300 mg/kg group effect was comparable to the vitamin E treatment group (Figure 3d,e).

### 2.9. BiE Treatment Restores Cardiac Tissue Histology

**Figure 4:**
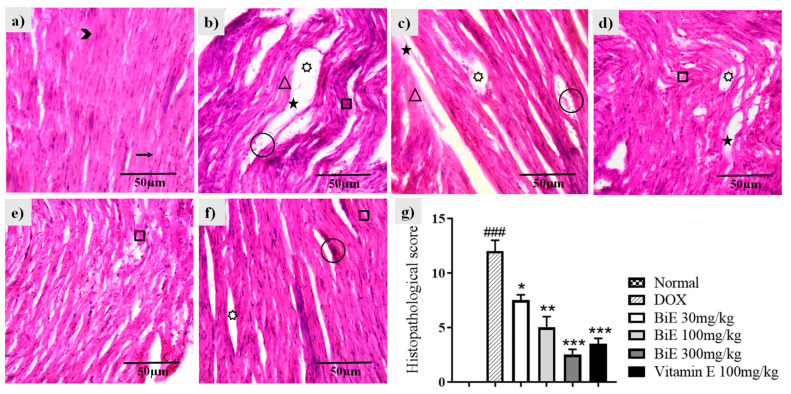
Histological changes in cardiac tissue following BiE and vitamin E treatment in DOX-induced cardiotoxicity (40×, scale bar = 50 µm). Representative H&E-stained sections from: (**a**) Normal, (**b**) DOX, (**c**) BiE 30 mg/kg, (**d**) BiE 100 mg/kg, (**e**) BiE 300 mg/kg, (**f**) vitamin E 100 mg/kg groups, and (**g**) histopathological scoring, where data (mean ± SEM) was analyzed using one-way ANOVA with Tukey’s post hoc test. Significance between the DOX and Normal groups is indicated as ^###^ = *p* < 0.001. In contrast, significance between the BiE or vitamin E groups and the DOX group is indicated by asterisks, denoted as * = *p* < 0.05, ** = *p* < 0.01, and *** = *p* < 0.001. Annotations: arrow = normal cardiac fibers; arrowhead = myocardial nuclei; square = wavy or fragmented fibers; star = tissue edema; triangle = inflamed myocytes; circle = vascular congestion; seven-point star = intracellular vacuolation.

Hematoxylin and eosin (H & E) staining of normal heart tissue showed cylindrical, dense cardiomyocytes with nuclei (Figure 4a). Doxorubicin administration disrupted cardiac architecture, resulting in marked alterations (Figure 4a). Disorganized, de-shaped, and broken cardiac fibers were seen in the DOX group (Figure 4b). Edema and congestion were also observed, along with decreased cardiomyocyte density. These changes depicted myocyte injury caused by doxorubicin when compared with the normal group. BiE and vitamin E treatments ameliorated these histopathological alterations, as shown in Figure 4c–f. A dose-dependent decrease in semi-quantitative scoring is shown in Figure 4g.

## 3. Discussion

Doxorubicin is a naturally occurring anthracycline chemotherapeutic drug with cardiotoxic adverse effects [25]. Among several mechanisms, oxidative stress and inflammation are considered the major pathways involved in DIC. Ongoing synthesis of potent ROS overwhelms the antioxidant capacity of heart tissue, leading to oxidative stress and cardiomyocyte damage. So, naturally occurring antioxidants and medicinal plants with antioxidant and anti-inflammatory activities are being investigated to prevent/delay DIC [26]. *Bassia indica* (Wight) A.J. Scott is a medicinal plant traditionally used for cardiac ailments in several countries, including Pakistan [14,15], Egypt [27], Saudi Arabia [18], and in Traditional Chinese Medicine [28]. It contains several biologically active phytoconstituents with anti-inflammatory activities [17,24]. We explored the cardioprotective effects of *B. indica* in the context of DIC and the mechanisms underlying cardiomyocyte protection.

GC-MS and HPLC analysis of BiE indicated the presence of flavonoid and phenolic compounds, including 2-Methoxy-4-vinylphenol, ferulic acid, gallic acid, quercetin, kaempferol, and coumaric acid. These phytoconstituents can arrest free radicals and restore endogenous antioxidant enzymes, providing a mechanistic basis for the potent antioxidant activity observed in BiE [28]. Antioxidant findings from BiE suggest that the observed phytochemical abundance is sufficient to account for the biological responses recorded. Antioxidant assays, including DPPH, FRAP, nitrite scavenging, H_2_O_2_ free radical scavenging, and CUPRAC, were performed to assess the antioxidant potential of BiE, which has NO and H_2_O_2_ free radical scavenging activity. It is vital in the context of DIC, which is mediated by increased levels of nitric oxide and hydrogen peroxide. The FDA approved dexrazoxane for the management of DIC, which reduces cardiac oxidative stress by chelating iron and, in turn, reducing the generation of iron-mediated free radicals [29]. BiE showed good reducing activity in the FRAP assay, thus decreasing the likelihood of iron-mediated free radical generation. Other antioxidant assays, including CUPRAC and DPPH, also showed good activity, indicating BiE’s free radical-scavenging efficacy (Table 3).

Doxorubicin administration increased the levels of several cardiac markers, including cTnI, CK-MB, LDH, and AST, compared to the normal group (Figure 1). The elevation of these markers in the DOX group reflects cardiomyocyte membrane disruption. Histomorphology of cardiac tissue showed damage to cardiomyocytes, with disorganized and fragmented cardiac fibers, along with inflammation, edema, and blood extravasation (Figure 4). Previous studies also reported a similar damage pattern after DIC, mediated through ROS production, lipid peroxidation, and mitochondrial damage. These cardiac markers were released from the heart following DIC-associated cardiac tissue injury [30,31]. BiE-treatment significantly reduced (*p* < 0.001) the serum levels of these cardiac markers, and disrupted and degenerated myofibrils compared to the DOX group, indicating that BiE treatment attenuated cardiac damage. The decrease in cardiac biomarkers was aligned with the restoration of cardiac architecture. Further, doxorubicin administration significantly increased MDA levels and decreased GSH, CAT, and SOD levels in the DOX group compared to normal heart tissues (Figure 2). A Similar kind of oxidative stress has been reported previously, where doxorubicin increased lipid peroxidation and decreased cellular enzymatic and non-enzymatic antioxidants [32,33]. Doxorubicin metabolism produces potent free radicals that cause lipid peroxidation in cardiomyocytes and, consequently, increase MDA levels in heart tissue. It also disrupts the balance between cellular oxidative and antioxidant pathways, depleting endogenous antioxidants such as SOD, CAT, and GSH [34]. This increased cardiac oxidative stress, with exhausted cellular antioxidant defenses, leads to myocardial injury, which, if managed earlier, can prevent the excessive damage [35]. BiE treatment decreased tissue MDA levels and increased endogenous SOD, CAT, and GSH levels in cardiomyocytes, indicating that BiE pretreatment can attenuate excessive oxidative stress induced by doxorubicin metabolism. Vitamin E also showed effects similar to those of BiE 300 mg/kg. These observed effects were also supported by histomorphology, which showed reduced inflammation and normal cardiac tissue architecture in BiE treatment groups (Figure 4). Some previous reports have also documented a similar cardioprotective mechanism in medicinal plants against doxorubicin-induced oxidative stress [36,37]. The increase in endogenous antioxidant enzymes and decrease in cardiac tissue degeneration are also directly linked to a reduction in cardiac biomarkers.

The Keap1/Nrf2 pathway is a major regulator of the cellular antioxidant system, and Nrf2 is an important transcription factor in this pathway. Keap1 enhances Nrf2 ubiquitination and degradation, whereas its absence promotes Nrf2 activity and increases antioxidant enzyme gene expression [38]. So Keap1 is being targeted to mitigate the oxidative cellular damage. We observed a significant upregulation of Keap1 and resulting downregulation of Nrf2 gene expression in diseased animals compared to the normal animals. Decreased Nrf2 levels compromise cardiomyocyte ability to cope with oxidative damage by weakening antioxidant pathways after doxorubicin exposure, thereby augmenting cardiomyocyte injury [39]. We observed a decrease in Keap1 gene expression, accompanied by increased Nrf2 mRNA levels, after BiE/vitamin E treatment. Several studies have reported that medicinal plant-based phytoconstituents can upregulate Nrf2 mRNA expression in DIC [40,41]. Nrf2 regulates the expression of many antioxidant enzymes via the antioxidant response element (ARE), including SOD, CAT, HO-1, etc., and plays a pivotal role in reducing oxidative stress. [42]. Significant upregulation of Nrf2 and reduction in Keap1 expression in BiE-treated rats suggest that BiE restored redox homeostasis by promoting Nrf2 nuclear translocation. This leads to increased expression of downstream antioxidant genes, consistent with the observed rise in SOD, CAT, and GSH levels.

Oxidative stress and inflammation are inextricably linked in the progression of DIC, as a significant increase in pro-inflammatory markers such as TNF-α and IL-1β has been reported with increased DOX-generated ROS [5]. We also observed a significant elevation in TNF-α and IL-1β levels after doxorubicin exposure compared with the normal heart. BiE treatment reduced the mRNA levels of these elevated inflammatory markers and increased IL-10 mRNA expression compared with the DOX group (Figure 3). Similar effects were also observed by other researchers, who reported that *Boesenbergia rotunda* and Danshensu treatment decreased DOX-induced inflammatory markers and protected heart tissue from injury by mitigating inflammation [36,41].

The effects of BiE were compared with those of vitamin E, and it was observed that although vitamin E produced a greater increase in endogenous antioxidant enzyme levels, BiE showed more prominent cardioprotective effects. This difference may be due to differences in their underlying mechanisms of action. Vitamin E functions mainly as an antioxidant, whereas *B. indica* exerts broader activity by simultaneously reducing oxidative stress, modulating the Keap1/Nrf2 pathway, and suppressing inflammation [24]. These multi-targeted actions, induced by phytochemicals present in the BiE, likely account for its comparable or superior effects.

## 4. Materials and Methods

### 4.1. Chemicals

Methanol, n-hexane, dichloromethane, n-butanol, ethyl acetate (Merck, Darmstadt, Germany), potassium ferricyanide, sodium nitrite, ferric chloride, EDTA, sodium carbonate (BDH, Laboratory Supplies, Poole, UK), DPPH, ethanol, hydrogen peroxide, trichloroacetic acid, ascorbic acid, hydrochloric acid (Sigma-Aldrich, St. Louis, MO, USA), neocuproine, tertamethoxypropane, thiobarbituric acid, nitrotetrazolium blue, HAC, (Macklin, Shanghai, China), cardiac troponin I, TNF-α, IL-1β, IL-10 ELISA kit (Nanjing Pars Biochemical, Nanjing, China), CKMB, LDH, AST kits (BSM, Madrid, Spain) keap-1, Nrf2 (SunLong Biotech Co., Ltd., Hangzhou, China), GSH kit (Solarbio Life Sciences, Beijing, China), ammonium molybdate tetrahydrate (Riedel-deHaen, Seelze, Germany).

### 4.2. Plant Collection and Extraction

The aerial parts (leaves, branches, and stem) of the *Bassia indica* plant were harvested (10 kg) with the help of a botanist (voucher no. 324/Botany) from district Muzaffargarh [31°10′5″ N 70°50′25″ E], Pakistan, and dried under shade. Plant powder (3 kg) was soaked in 70% methanol (15 L) for 7 days with occasional shaking and then filtered. The same procedure was repeated twice with the filtrate residue, and the entire filtrate was evaporated in a rotary evaporator to obtain a semisolid *Bassia indica* extract (BiE). A portion of BiE (150 g) was fractioned sequentially with solvents (250 mL each) of increasing polarity, including n-hexane, dichloromethane, n-butanol, ethyl acetate, and water. All fractions were stored at −20 °C [19].

### 4.3. GC-MS Analysis

GC-MS of BiE was performed as previously described [19]. A Thermo Scientific (DSQI) GC, configured with the NIST20.L library, was used. Compounds were identified by comparing peak, retention time, and spectral patterns with those in the NIST20.L library. Detailed conditions of GC-MS are tabulated in Supplementary Table S1.

### 4.4. HPLC Analysis

For the detection and estimation of phenolic and flavonoid compounds in BiE, gradient HPLC was performed using an LC-10A (Shimadzu, Kyoto, Japan). BiE 50 mg was homogenized in 24 mL of methanol, followed by the addition of 10 mL HCL (6M) and 16 mL of HPLC-grade H_2_O. This mixture was refluxed for 2 h and filtered. The detailed HPLC conditions are given in Supplementary Table S2. Compound identification was performed by comparing retention times and peak patterns with those of standards, while quantification was performed by external calibration [43,44].

### 4.5. Antioxidant Assays

#### 4.5.1. DPPH Scavenging Assay

DPPH (150 µL, 200 mM) was added to a 96-well plate along with 50 µL BiE and its fractions/ascorbic acid dilutions, and the mixture was incubated in the dark for 30 min at 25 °C. For control, 50 µL methanol was used. Absorbance was recorded at 517 nm. [45]. Percentage scavenging was determined using the standard formula, and IC_50_ values were subsequently calculated.

(1) $$Percent\;scavenging\;\left(\%\right)=\left(\frac{Abs.\;of\;control-Abs.\;of\;sample}{Abs.\;of\;control}\right)\times 100$$

#### 4.5.2. FRAP Assay

BiE and its fractions (1 mg/mL, 100 µL) were mixed with 250 µL of PBS (200 mM, pH 6.6) and 250 µL of 1% potassium ferricyanide. The mixture was incubated at 50 °C for 20 min, then 250 µL of 10% TCA was added, followed by centrifugation at 3000 rpm for 10 min. A 100 µL portion of the supernatant was transferred to a 96-well plate and diluted with an equal volume of distilled water, incubated for 10 min, and then 20 µL of 1% FeCl_3_ was added. Ascorbic acid (15.62–250 µg/mL) was used to construct the calibration curve, and PBS served as the blank. Absorbance was read at 700 nm. FRAP activity was expressed as µM AAE/g BiE [44].

#### 4.5.3. Nitric Oxide Assay

The NO scavenging assay was carried out as previously described [46]. BiE, its fractions, or ascorbic acid (1 mL; 0.078–10 mg/mL) was mixed with 1 mL of 1 mM NaNO_2_, and the pH was adjusted to 2 using 0.1 N HCl. The volume was then brought to 10 mL and incubated at 37 °C for 1 h. Subsequently, 100 µL of the reaction mixture was combined with 500 µL of distilled water, followed by the addition of 100 µL of Griess reagent. After a 15-min incubation at 25 °C, absorbance was measured at 540 nm with and without Griess reagent. A control was prepared using distilled water instead of the sample. Percentage scavenging was calculated using the standard formula, and IC_50_ values were determined.

$$\%age\;scavenging\;activity=100-\left[\left\{\frac{Abs.\;with\;GR-Abs.\;without\;GR}{Abs.\;of\;Control}\right\}\times 100\right]$$

#### 4.5.4. CUPRAC Assay

The CUPRAC assay was used to evaluate the antioxidant capacity of BiE and its fractions [44]. Equal volumes of 7.5 mM neocuproine solution in 96% ethanol, 10 mM CuCl_2_·2H_2_O, and 1 M ammonium acetate buffer (pH 7) were mixed to obtain the CUPRAC reagent. To the 96-well plate, 150 µL CUPRAC reagent and 150 µL BiE (0.5 mg/mL) were added, and the plate was incubated for 1 h. For the calibration curve, ascorbic acid (0.1–0.8 mM) was used. Absorbance was recorded at 450 nm after incubation and represented as mM AAE/g of BiE.

#### 4.5.5. H_2_O_2_ Scavenging Assay

H_2_O_2_ free radical scavenging activity was determined by adopting the previously described method with some modifications [47]. H_2_O_2_ (2 mM) was prepared in 50 mM PBS (pH 7.4). BiE and its fractions/ascorbic acid 100 µL (1000–62.5 µg/mL) of each sample were mixed with 300 µL PBS. Then, 600 µL H_2_O_2_ was added to all samples, and the samples were incubated for 10 min. Control reaction contained PBS and H_2_O_2_. After incubation, absorbance was measured at 230 nm against a blank. H_2_O_2_ free radical scavenging activity was calculated using the given equation, and IC_50_ values were determined.

$$Hydrogen\;peroxide\;scavenging\;activity\;=\left(\frac{Abs.\;of\;control-Abs.\;of\;sample}{Abs.\;of\;control}\right)\times 100$$

### 4.6. Doxorubicin-Induced Cardiotoxicity

#### 4.6.1. Animals

Animals were maintained under standard laboratory conditions as described previously [48]. They were acclimatized for two weeks before the start of the experiments. All experimental procedures were reviewed and approved by the Pharmacy Animal Ethics Committee (PAEC file no. PAEC/23/101).

#### 4.6.2. Study Design

Animals were randomly assigned to six groups, with five animals per group. The normal and intoxicated groups were given normal saline (4 mL/kg, p.o.) daily for 28 days. The treatment groups received BiE at doses of 30, 100, or 300 mg/kg, whereas the standard group was administered vitamin E (100 mg/kg, p.o.) for the same duration. Starting on day 13, all groups except the normal control were injected with doxorubicin (5 mg/kg, i.p.) every third day until the cumulative dose reached 25 mg/kg [49]. At the conclusion of the study, animals were anesthetized with a xylazine-ketamine mixture (1:10) for blood collection. Hearts were excised, rinsed with chilled saline, and divided into three sections. One section was fixed in 10% buffered formalin for histological examination, while the remaining tissues and serum samples were stored at −20 °C for subsequent biochemical and oxidative stress analyses.

#### 4.6.3. Assessment of Cardiac Markers

Serum cTnI concentrations were quantified using an ELISA kit following the manufacturer’s instructions (Nanjing Pars Biochemical, Nanjing, China). CK-MB, LDH, and AST activities were determined by colorimetric methods using the MicroLab 300 (Merck, Darmstadt, Germany), according to the manufacturer’s protocol (BSM, Madrid, Spain).

#### 4.6.4. Assessment of Inflammatory Markers by ELISA

Serum concentrations of TNF-α, IL-1β, and IL-10 were determined using ELISA kits, according to the manufacturer’s instructions (Nanjing Pars Biochemical, Nanjing, China). Keap1 and Nrf2 levels were quantified in homogenized heart samples using commercially supplied ELISA kits according to the manufacturer’s instructions (SunLong Biotech Co., Ltd., Hangzhou, China). After completing the assay steps, absorbance was recorded at 450 nm, and the concentrations of the respective biomarkers were calculated from the standard curves generated for each kit.

#### 4.6.5. Heart Homogenate Preparation

Heart tissues were weighed, cut into small pieces, and a 10% *w*/*v* tissue homogenate was prepared, and total protein contents were determined by the Bradford method.

#### 4.6.6. Malondialdehyde Assay

MDA levels in heart tissues were determined as described previously [46]. Heart tissue homogenate 100 µL was mixed with 200 µL ice-cold trichloroacetic acid (10%). The mixture was incubated on ice for 15 min, then centrifuged at 4 °C at 2200× *g* for 15 min to obtain the supernatant. Tissue sample supernatants and 200 µL TMP (0–100 µM) were mixed with an equal volume of 0.67% thiobarbituric acid and incubated for 10 min in boiling water. After cooling, absorbance was measured at 532 nm in a microplate reader, and MDA levels were calculated from the TMP standard curve.

#### 4.6.7. Glutathione Assay

Glutathione content in heart tissues was assessed with a commercially available kit according to the manufacturer’s protocol (Solarbio Life Sciences, Beijing, China). Absorbance was measured at 412 nm, and GSH in tissue samples was quantified from a GSH standard curve (12.5–200 µg/mL).

#### 4.6.8. Catalase Assay

CAT in heart tissues was assessed according to the previously described method [46]. A substrate consisting of 60 µM H_2_O_2_ in 60 mM PBS (pH 7.4) was used for this assay. Tissue homogenate (100 µL) was incubated with 1 mL substrate for 3 min at 37 °C, and then 4 mL of ammonium molybdate tetrahydrate (32.4 mM) was added to stop the reaction. For the standard, d.H_2_O (100 µL) was used instead of the homogenate, whereas the control contained only distilled water. The optical density (O.D.) of all samples was measured at 374 nm, and catalase enzyme activity was calculated using the given equation.

$$CAT\;=\frac{2.303}{t}\times \left[log\frac{S{}^{\circ}}{S-M}\right]\times \frac{Vt}{Vs}$$

#### 4.6.9. Superoxide Dismutase Assay

SOD activity in heart tissue homogenates was measured as described previously [44]. In a 96-well plate, 50 µL tissue homogenate was added, followed by 50 µL of sodium carbonate (50 mM), 20 µL of 0.1 mM EDTA, and 20 µL of 0.56 mM NBT. The reaction started by adding 40 µL of 1 mM HAC. Blank was prepared by adding sodium carbonate (60 µL), EDTA (30 µL), NBT (55 µL), and HAC (55 µL). Absorbance of samples was measured at 570 nm at 0, 5, 10, 15, and 20 min, and SOD activity was calculated using the given equations:

$$SOD\;activity=X=\frac{change\;in\;abs.\;of\;control-\;change\;in\;abs.\;of\;sample}{change\;in\;abs.\;of\;control}$$

$$SOD\;Units/mL=\frac{X}{50}⨯\frac{Reaction\;volume}{Sample\;volume}$$

$$SOD\;activity\;units/mg=\frac{SOD\;units/mL}{protein\;content}$$

#### 4.6.10. mRNA Expression of Inflammatory and Oxidative Stress Markers

The transcript levels of Keap1, Nrf2, TNF-α, IL-1β, and IL-10 were evaluated by quantitative real-time PCR (qRT-PCR). Total RNA was extracted using the HiPure RNA isolation kit, and complementary DNA (cDNA) was synthesized from the purified RNA with the ThermoFisher cDNA synthesis kit. qRT-PCR amplification was carried out using SYBR Select Master Mix on the SLAN-96P real-time PCR platform, employing the instrument’s standard thermal cycling program. Data collection and expression analysis were performed through the SLAN-96P multi-tasking software interface (version 7.0, Sansure Biotech, Changsha, China). GAPDH was used as the endogenous control for normalization of gene expression levels. All primers used are listed in Table S3 and were sequence verified before use.

#### 4.6.11. Histomorphology of the Heart

Formalin-fixed heart tissues were washed in running water for 8–12 h and dehydrated with 70–100% ethanol. Afterward, tissues were submerged in absolute xylene for 3 h, then embedded in paraffin wax. Tissue sections of 5–10 µm thickness were prepared using a microtome and subsequently stained with hematoxylin and eosin (H&E). The stained sections were examined under a camera-equipped light microscope at 40× magnification, and images were captured using PixelPro 3.0 software. Histopathological evaluation focused on identifying inflammation, neutrophil infiltration, edema, and other markers of tissue injury [24].

### 4.7. Statistical Analysis

All experimental data were processed and analyzed using GraphPad Prism version 10 (GraphPad Software, San Diego, CA, USA). Results are expressed as mean ± SEM. Differences among groups were evaluated using one-way ANOVA, followed by Tukey’s post hoc multiple comparison test to determine significance. A *p*-value of less than 0.05 (*p* < 0.05) was regarded as statistically significant.

## 5. Conclusions

*Bassia indica* exerts cardioprotective effects against DIC by suppressing oxidative stress and inflammation through modulating the Keap1/Nrf2 signaling pathway. BiE contained several important phytochemicals, including 2-Methoxy-4-vinylphenol, ferulic acid, gallic acid, quercetin, kaempferol, and coumaric acid. These compounds are potent antioxidants that prevent lipid peroxidation and oxidative stress-induced cardiac injury by enhancing the endogenous SOD, CAT, and GSH antioxidant enzyme activity. These findings were supported by reduced cardiac injury markers and improved cardiac tissue architecture observed in histological analysis. These results validate the traditional uses of *B. indica* as a natural cardioprotective agent; however, further studies are required to provide mechanistic insight and to evaluate its use as a promising adjuvant therapy for drug-induced cardiotoxicity.

## 6. Limitations of the Study

This study has certain limitations. We kept the sample size to five animals per group; larger cohorts may further strengthen the statistical robustness. Molecular docking and pharmacokinetic profiling were not conducted, and bioactive constituents were not isolated to correlate biological activity with mechanistic insight; future studies will address these aspects.

## Figures and Tables

**Table 1:** Phytochemicals identified in GC-MS analysis of BiE.

RT	Area	Compound Name	M.F.	M.W.
7.10	2.39	2-Methoxy-4-vinylphenol	C_9_H_10_O_2_	150.1
11.09	2.01	Megastigmatrienone	C_13_H_18_O	190.2
12.91	2.64	Hexadecanoic acid, methyl ester	C_17_H_34_O_2_	270.4
13.10	6.33	*(E)*-4-(3-Hydroxyprop-1-en-1-yl)-2-methoxyphenol	C_10_H_12_O_3_	180.2
13.50	11.09	n-Hexadeconic acid	C_16_H_32_O_2_	256.4
13.87	1.31	Loliplode	C_11_H_16_O_3_	196.2
13.95	1.51	Benzenepropanoic acid, 3,5-bis(1,1-dimethylethyl)-4-hydroxy-, methyl ester	C_18_H_28_O_3_	292.4
14.76	1.61	*(E)*-9-Octadecenoic acid, methyl ester	C_19_H_36_O_2_	296.4
14.84	2.81	9,12-Octadecadienoic acid *(Z,Z)*-, methyl ester	C_19_H_34_O_2_	294.4
15.04	4.04	9,12,15-Octadecatrienoic acid, methyl ester, *(Z,Z,Z)*-	C_19_H_32_O_2_	292.4
15.34	3.83	Oleic acid	C_18_H_34_O_2_	282.4
15.43	7.21	10-*(E)*,12-*(Z)*-Octadecadienoic acid	C_18_H_32_O_2_	280.4
15.63	5.18	9,12,15-Octadecatrienoic acid	C_18_H_30_O_2_	278.4
15.93	3.93	*trans*-Sinapyl alcohol	C_11_H_14_O_4_	210.2
18.15	1.17	Phenol, 2,2′-methylenebis [6-(1,1-dimethylethyl)-4-methyl-	C_23_H_32_O_2_	340.5
18.26	3.66	Hexadecanoic acid, 2-hydroxy-1-(hydroxymethyl)ethyl ester	C_19_H_38_O_4_	330.5
18.37	1.39	1,3,5-triphenylcyclhexane	C_24_H_24_	312.4
18.68	2.88	*L*-Tryptophan, *N*-acetyl-, methyl ester	C_14_H_16_N_2_O_3_	260.2

RT: retention time; M.F.: molecular formula; M.W.: molecular weight.

**Table 2:** Bioactive phytochemicals of BiE identified in the HPLC assay.

Compound	Retention Time (Min)	Area (%)	Quantification (ppm)
Quercetin	3.3	0.7	4.6
Gallic acid	4.9	0.6	3.2
Caffeic acid	12.8	0.3	1.7
Syringic acid	13.6	0.4	1.3
Benzoic acid	14.8	2.1	27.1
Chlorogenic acid	15.5	2.0	19.1
p-Coumaric acid	17.4	2.5	3.9
m-Coumaric acid	20.3	1.8	1.1
Ferulic acid	22.0	1.0	8.7
Kaempferol	9.2	69.3	31.4

**Table 3:** Antioxidant assays of BiE and its fractions.

Fraction	DPPH IC_50_ (µg/mL)	NO IC_50_ (mg/mL)	H_2_O_2_ IC_50_ (µg/mL)	FRAP (μmoL AAE/g)	CUPRAC (mg AAE/g)
n-hexane	203.6 ± 50.3	1.7 ± 0.1	185.8 ± 4.6	20.3 ± 3.1	27.6 ± 2.6
DCM	18.2 ± 8.0	1.7 ± 0.2	133.1 ± 6.6	53.7 ± 13.5	120.7 ± 5.5
n-butanol	37.4 ± 19.5	0.17 ± 0.008	203.3 ± 7.0	81.5 ± 9.9	122.7 ± 1.7
EA	12.6 ± 2.6	0.36 ± 0.007	189.1 ± 4.8	110.2 ± 14.6	126.9 ± 0.2
Aqueous	136.9 ± 19.2	7.5 ± 0.3	193.2 ± 7.6	33.9 ± 4.1	69.0 ± 3.9
BiE	45.2 ± 2.1	3.9 ± 0.1	161.5 ± 4.2	119.6 ± 5.2	113.3 ± 5.9
Ascorbic acid	11.9 ± 3.8	0.46 ± 0.04	128.7 ± 7.8	Nd	Nd

Nd: Not determined.

## Data Availability

Data is from F.A.’s Ph.D. thesis and can be provided at a reasonable request. The data are not publicly available due to the requirement of Thesis submission to the Higher Education Commission of Pakistan.

## Supplementary Materials

The following supporting information can be downloaded at https://www.mdpi.com/article/10.3390/ph18121907/s1. GC-MS chromatogram of hydro alcoholic *B. indica* extract; Figure S1: GC-MS chromatogram of BiE. Figure S2: HPLC chromatograms of BiE; Table S1: Conditions of GC-MS; Table S2: HPLC gradient. Table S3: Primer sequence of qPCR analysis for BiE.
