# Supplementary material for: Bassia indica Attenuates Cardiotoxicity in a Rat Model via Anti-Inflammatory, Antioxidant, and Keap1/Nrf2 Modulation
Source: Pharmaceuticals (Basel). 2025 Dec 18;18(12):1907. doi: 10.3390/ph18121907 (PMC12736070; doi:10.3390/ph18121907)
Supplement: Supplementary file 1 [file pharmaceuticals-18-01907-s001.zip › pharmaceuticals-3986070-supplementary.pdf]

Bassia indica Ameliorates Doxorubicin-Induced Cardiotoxicity by Suppressing the Oxidative Stress

Table S1: Conditions of GCMS

Parameter	Conditions
Capillary column	TR-5MS
Column length	30 M
Column diameter	0.25 mm
Film thickness	0.25 μm
Carrier Gas	Helium
Flow rate	1 mL/min
Sample volume	1 μL
Injector temperature	250 °C
Oven temperature	50-300 °C
Temperature rate	15 °C/min

Table S2: HPLC gradient

Parameter	Conditions
Column	CLC-ODS
Column specifications	25cm x 4.6mm, 5m
Sample volume	20μl (10mg/ml)
Sampler	SIL-20A auto-sampler
Mobile phase solvent A	H_2O:CH3COOH-94:6, pH 2.2
Mobile phase solvent B	CH_3CN
Elution rate	1.0 ml/min
Absorbance	280 nm
Detector	SPD-10AV UV/VIS

Table S3. Primer sequence of qPCR analysis for BiE.

Gene	Primer Sequence (5→3)	Forward/Reverse (F/R)
**KEAP1**	CCCTGTGCCTCTATGAGCGT	F
	TGCCACTCGTCTCGATCTGG	R
NRF2	CCCAGCACATCCAGACAGACA	F
	GGCTGGGAATATCCAGGGCAA	R
**GAPDH**	G ACTCCACTCACGGCAAATTC	F
	TCTCCATGGTGGTGAAGACA	R
**IL-10**	AAGGCAGTGGAGCAGGTGAA	F
	CCAGCAGACTCAATACACAC	R
**IL-1b**	TGGACCTTCCAGGATGAGGACA	F
	GTTCATCTCGGAGCCTGTAGTG	R
**TNF-α**	ATGGGCTCCCTCTCATCAGT	F
	GCTTGGTGGTTTGCTACGAC	R

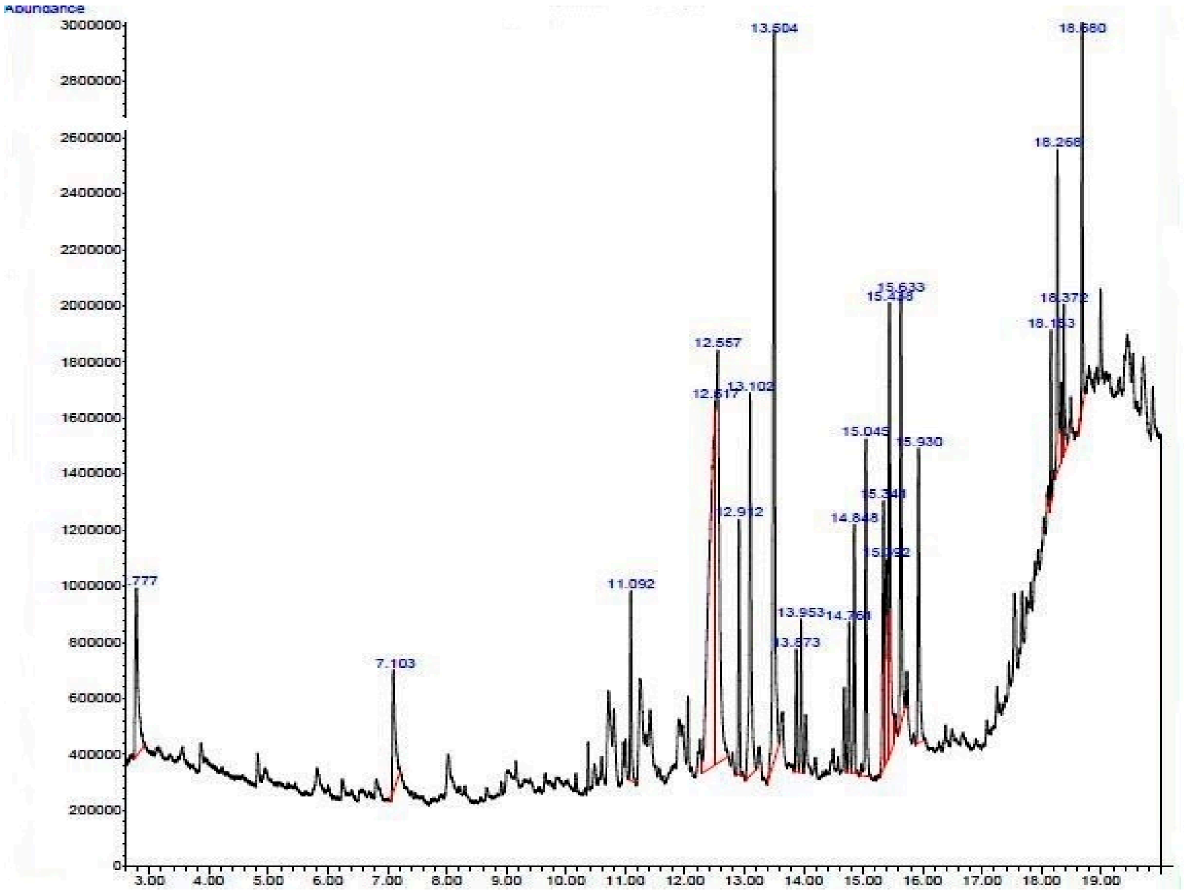

Figure S1. GC-MS chromatogram of BiE

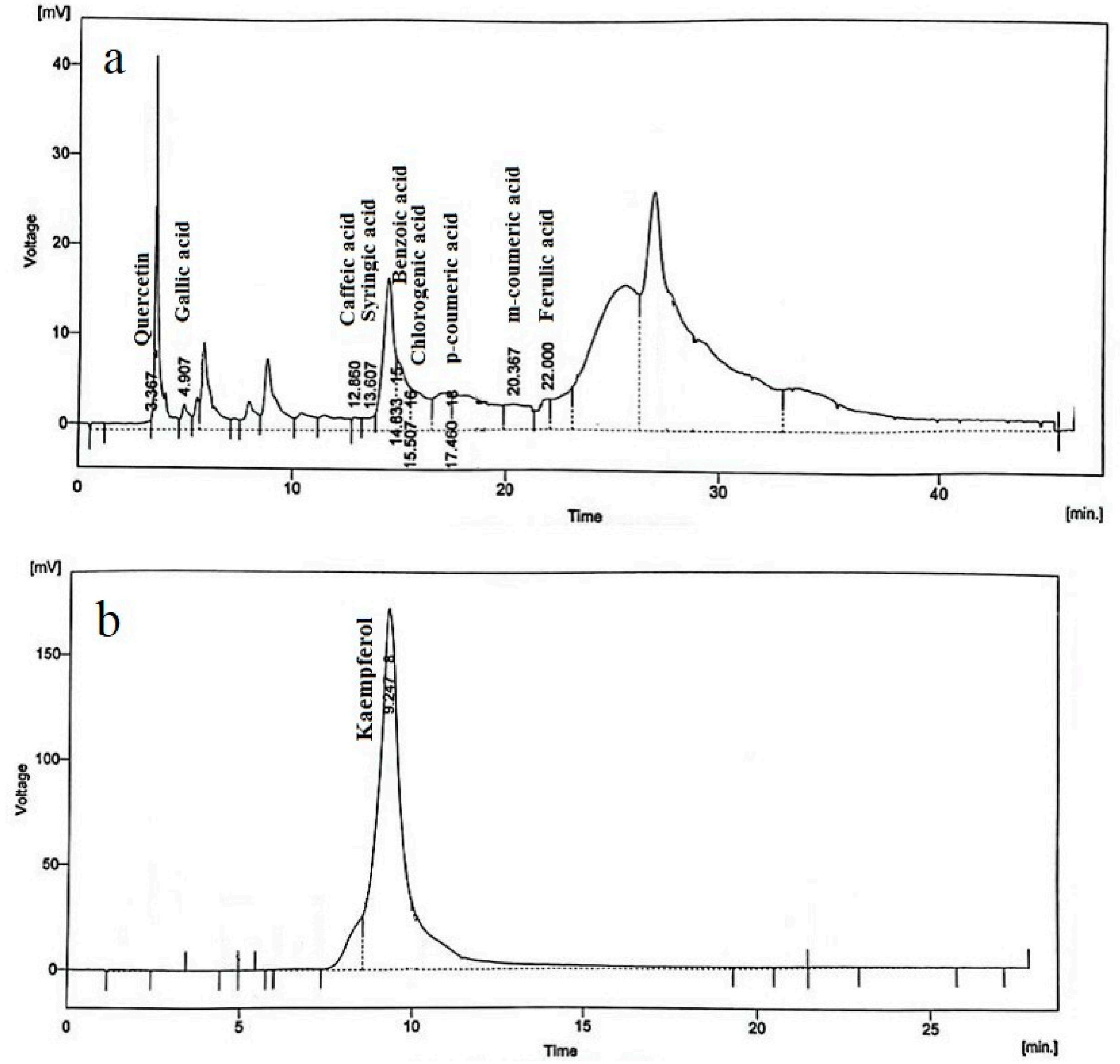

Figure S2. HPLC chromatograms of BiE
